# Vortex fluidic mediated encapsulation of functional fish oil featuring in situ probed small angle neutron scattering

**DOI:** 10.1038/s41538-020-00072-1

**Published:** 2020-09-09

**Authors:** Shan He, Nikita Joseph, Marzieh Mirzamani, Scott J. Pye, Ahmed Hussein Mohammed Al-anataki, Andrew E. Whitten, Yaonan Chen, Harshita Kumari, Colin L. Raston

**Affiliations:** 1 Department of Food Science and Engineering, School of Chemistry Chemical Engineering, Guangzhou University, Guangzhou, 510006 China; 2 Flinders Institute for Nanoscale Science and Technology, College of Science and Engineering, Flinders University, Bedford Park, SA 5042 Australia; 3 Division of Pharmaceutical Sciences, James L. Winkle College of Pharmacy, University of Cincinnati, Cincinnati, OH 45267-0004 USA; 4 Australian Nuclear Science and Technology Organisation (ANSTO), New Illawarra Road, Lucas Heights, NSW 2234 Australia

**Keywords:** Biophysical chemistry, Polysaccharides

## Abstract

Major challenges for optimizing the benefits of fish oil on human health are improved bioavailability while overcoming the strong odor and avoiding significant oxidation of the omega-3 polyunsaturated fatty acids (PUFAs). The scalable continuous flow thin film vortex fluidic device (VFD) improves the Tween 20 encapsulation of fish oil relative to conventional homogenization processing, with the fish oil particles significantly smaller and the content of the valuable omega-3 fatty acids higher. In addition, after 14 days storage the remaining omega-3 fatty acids content was higher, from ca 31.0% for raw fish oil to ca 62.0% of freeze-dried encapsulated fish oil. The VFD mediated encapsulated fish oil was used to enrich the omega-3 fatty acid content of apple juice, as a model water-based food product, without changing its sensory values. The versatility of the VFD was further demonstrated in forming homogenous suspensions of fish oil containing water-insoluble bioactive molecules, curcumin and quercetin. We have also captured, for the first time, real-time structural changes in nanoencapsulation by installing a VFD with in in situ small angle neutron scattering. Real-time measurements afford valuable insights about self-assembly in solution.

## Introduction

Fish oil is an excellent dietary source of omega-3 polyunsaturated fatty acids (PUFAs) having positive effects on human health. However, it has a strong odor and is easily oxidized at ambient conditions^1^ and omega-3 PUFAs cannot be synthesized in the human body, and thus it needs to be sourced from food^2^. Capsules of fish oil protect omega-3 PUFAs from oxidation, but their large size limits their utility as nutritional supplements. Encapsulating fish oil in nanoparticles also protects omega-3 PUFAs from oxidation, and has exciting possibilities in food processing. Although such confinement of one substance (active agent) within another (wall material)^3^ has been broadly studied for fish oil^4,5^, the methods used either require multiple steps or expensive processing equipment.

**Fig. 1:**
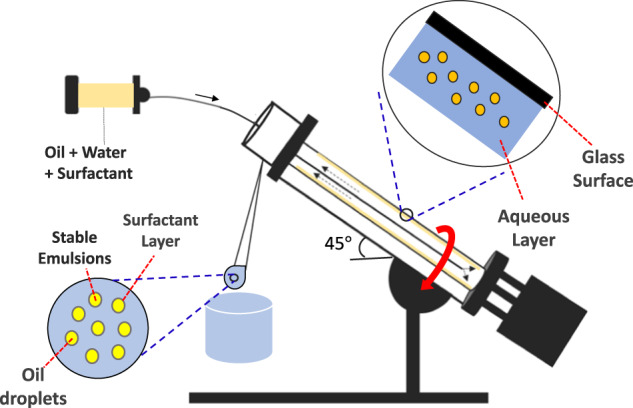
The vortex fluidic device (VFD). Schematic of the vortex fluidic device (VFD) in generating emulsions, with a jet feed delivering liquid at a certain flow rate to the rapidly rotating borosilicate glass or quartz tube (typically 20mm OD, 17.5mm ID), with the liquid as a mixture of water, fish oil, and Tween 20 driven up the tube tilted at 45° relative to the horizontal position (*θ*).

The vortex fluidic device (VFD) (Fig. 1) is a continuous flow thin film microfluidic platform with diverse applications^6^. It harnesses high shear forces, intense micro mixing, and high mass transfer to overcome the mixing and heat transfer limitations akin to traditional batch processing^7^. Processing capabilities of the VFD are rapidly growing across various fields, ranging from small-molecule synthesis involving a number of steps being accomplished in a single pass to processing functional materials for drug delivery and manipulating single-cell organisms^8^. Enzymatic reactions in the VFD have on average a sevenfold acceleration in their catalytic reactions^9^, depending on the choice of processing parameters of the device, including rotation speed of the inclined glass tube^9^. Tethering enzymes to the surface of the glass tube has also been achieved for the synthesis of complex molecules in a single pass under continuous flow conditions^10^. Fabricating nanomaterials in the VFD has yielded unexpected results, also under continuous flow, for example, in assembling fullerene C_60_ molecules into nano-tubules with hollow diameters of 100–400 nm, in the absence of a surfactant and without the need for further downstream processing^11^. Other noteworthy applications of the VFD are converting sunflower oil to biodiesel at room temperature with no saponification and avoiding the conventional use of co-solvents or complex catalysts^12^, and the direct transesterification of microalgae^13^.

As a preliminary trial, our previous study^14^ established that for low concentrations of the fish oil (fish oil: surfactant = 1:1 (w/w), fish oil + surfactant:water = 2 mg/ml), the VFD is effective in encapsulating fish oil particles below 300 nm, whereas those produced by homogenization are 3–4 µm in diameter. Though the low concentration in this study was not industry-applicable because of the high cost of concentrating and drying the material, this proof-of-concept achievement encouraged us to follow up with a high concentration study, to drastically reduce the cost of the overall processing. In the present study, we report a simple low-cost, single pass VFD process for encapsulating fish oil using high concentration feedstocks (fish oil: surfactant = 1:1(w/w), fish oil + surfactant:water = 0.2 g/ml), as well as the ability to simultaneously encapsulate sparingly water-soluble bioactive polyphenols. The encapsulated omega-3 fatty acids become protected from oxidation, and the encapsulation is effective in enriching the omega-3 fatty acid content in liquid food. Also reported is a small-angle neutron scattering (SANS) method for studying the shear stress-induced encapsulation process under real time conditions in the VFD. Notably post-VFD SANS studies have been reported^15^, however, real-time measurement of self-assembly in solution is being reported here for the first time.

## Results

The oil-in-water encapsulations had a surfactant-to-oil ratio of 1:1 (w:w), and a total concentration of 0.2 g/mL (10 mL of the Tween 20 oil suspension in water was premixed as pretreatment for both VFD processing and the control of homogenization processing, which was composed of 1 g of oil, 1 g of Tween 20, and 8 mL of water. The VFD was operated at optimized conditions, where the rotation speed was 9000 rpm, flow rate 0.3 mL/min, tilt angle 45°, and 25 °C. The tilt angle of 45° has been reported as the optimal angle in many previous studies regarding VFD operation^9,10,14,16,17^. Our previous study^14^ regarding fish oil encapsulation at low concentration also reported that the particle size of encapsulated fish oil was the smallest when the tilt angle was set at 45° while operating the VFD, as measured by dynamic light scattering (DLS). Tween 20 was selected as the surfactant due to its food grade category and long-chain character, which are two fundamental requirements for fish oil encapsulation in high concentration for food application.

**Fig. 2:**
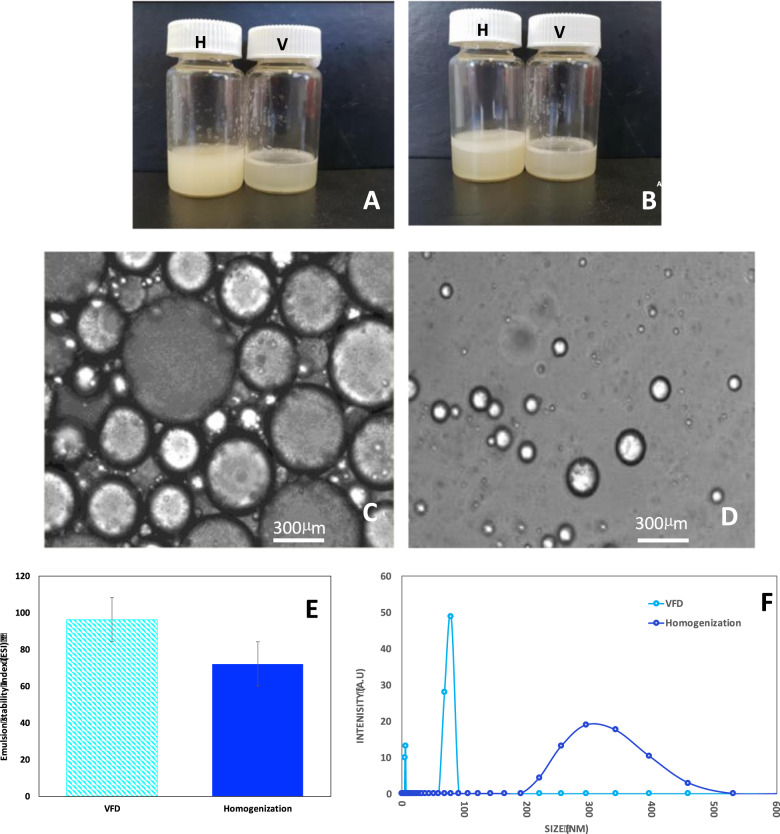
Fish oil encapsulation with Tween 20. **a** Homogenization processed mixture (speed: 13,500 rpm; time: 10 min; temperature: 25 °C) immediately after processing (H) and VFD processed mixture (rotational speed: 9000 rpm; flow rate: 0.3 mL/min; tilt angle: 45°; temperature: 25 °C) immediately after processing (V). **b** The mixtures in (**a**) after 24 h. **c**, **d** Epi-fluorescence microscopic images (20× long-working-distance (20× amplification); exposure time 100 ms; frame rate 10 fps) for the homogenization and VFD processed mixtures in (**a**), respectively. **e** Emulsion stability of homogenization and VFD processed mixtures in (**a**). **f** Dynamic light scattering (temperature: 25 °C; He–Ne wavelength: 633 nm detector angle: 173 °C) results of homogenization and VFD mixtures prepared in (**a**).

Figure 2a, b shows the appearance of the fish oil encapsulation solution immediately after processing and 24 h later, respectively. While both samples were homogeneous immediately after processing, the sample prepared using homogenization phase separated after 24 h, whereas that prepared using VFD processing was stable over the same period. The encapsulation stability of the immediately prepared mixture (Fig. 2e) processed using the VFD (96%) was significantly higher than that processed using homogenization (72%). These results were in accordance with the images shown in Fig. 2a, b and reflect the appearance of white oily foam on top of encapsulation liquid. The combination of these results demonstrated that in comparison with conventional method of homogenization, the VFD method is more efficient to form stable emulsions. Epi-fluorescent microscopy, Fig. 2c, d, clearly establishes that the particle sizes of the encapsulation sample processed by homogenization were much larger than those of the sample processed by VFD, and this comparison was further complimented by DLS data (Fig. 2f). Particles of VFD-encapsulated fish oil were much smaller than that of homogenization-encapsulated fish oil (Fig. 2f). Indeed, due to the accuracy of current DLS technique, DLS results were better considered as guidance, but both DLS results and Epi-fluorescent microscopic results demonstrated the dramatic size difference between the samples produced by homogenization and VFD. In comparison with particle sizes of samples produced by homogenization, the particle sizes of samples produced by VFD are drastically reduced. The difference between encapsulated particle size using VFD and homogenization processing for different lipid surfactants was similar to that for low concentrations of lipid^14^. Furthermore, VFD processing is able to provide continuous processing, whereas homogenization is only able to provide batch processing. Considering the industrial production regarding cost of operation, labor, etc., using continuous process is more favorable for industry. To establish this herein, we translated the processing of a 10 mL solution, as used for all of the above studies, to passing 100 mL of solution within 18 h through one device. The resulting solution had the same overall characteristics, including colloidal stability. The success of this trial demonstrates the stability of VFD processed material, and the consistency of the quality in the final products, for translating into even larger scale processing involving a number of devices operating simultaneously. It is also noteworthy that a high-pressure homogenization system with microjet feed might also be able to generate similar results to that of the VFD. However, the high cost of this system (approximately USD 150 k) limits its application mainly to laboratory processing. In contrast, a VFD system is relatively inexpensive and is applicable for both laboratory and industry applications, with the cost of one VFD device USD 15 K. Thus for the cost of purchasing one high-pressure homogenization system, 10 VFD can be purchased with much larger production capacity when operating under continuous flow in parallel.

The energy consumptions of the VFD and homogenizer have also been considered. For the VFD operating at 9000 rpm (optimal rotational speed), the energy input is 232 w and given that it takes 7 min for the first drop of liquid to be collected, for then 35 min of processing to pass 10 mL of solution through the device at of 0.3 mL/min (optimal flow rate), the total energy consumption is 162.4 wh. The energy input of the homogenizer (T25 digital ULTRA-TURRAX) is 800 w and for 10 min processing of 10 mL of solution (optimal processing parameters), the energy consumption is 136 wh. Clearly the energy consumption for 10 mL of liquid is less in the homogenizer, but under continuous flow processing in the VFD, the energy consumption for this device becomes competitive; passing 420 mL of liquid through the VFD consumes 232 w × [(7 min + (35 min × 42))/60] h = 5711 wh, whereas for batch processing in the homogenizer for the same volume of liquid, the energy consumption is 136 wh × 42 = 5712 wh. Thus, in terms of energy consumption, passing even larger volumes through the VFD will make it an attractive processing platform over the homogenizer. This coupled with other attributes of continuous flow processing in the VFD makes it attractive for industrial processing relative to homogenization.

**Fig. 3:**
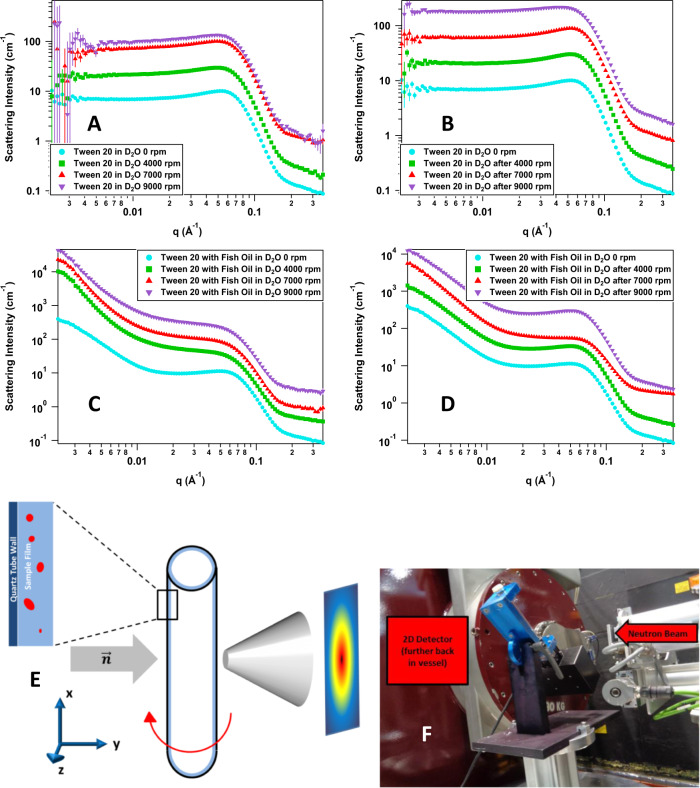
Small-angle neutron scattering. **a** SANS data overlay of real-time VFD data of 10 wt% Tween 20 alone, **b** SANS data overlay of post-VFD data of 10 wt% Tween 20 alone, **c** SANS data overlay of real-time VFD data of 10 wt% Tween 20 with 10 wt% of fish oil, **d** SANS data overlay of post-VFD data of 10 wt% Tween 20 with 10 wt% of fish oil, **e** top-down view schematic representation for in situ VFD SANS where the neutron beam is normal to the *x*–*z* plane of the VFD tube and the tube is tilted 45° from vertical, **f** picture of the VFD installed on the sample staging area of the Bilby beam line while out of the path of the neutron beam. The curves shown in the data overlays in **a**–**d** were offset by powers of 3 to improve clarity.

For a better understanding at the nano-dimensions, a new small-angle neutron scattering (SANS) setup and study is carried out in-presence of the VFD. SANS is a powerful technique capable of elucidating the size and geometry of objects on the mesoscopic scale (1–100 nm), making it well-suited to studying soft matter systems such as micelles. The large size of the SANS instrument’s sample staging area also allows the sample setup to be changed in order to study the effects of a stimulus on the system size and geometry in real-time. One example of this versatility is rheo-SANS^18^, where a rheometer is placed in the sample area and a sample is sheared during the SANS experiment to study how the system responds to shear. Other setups, such as high-pressure and dielectric spectroscopy, have also been used in combination with SANS. Because of this versatility, SANS was chosen to study the real-time effects of the VFD on the size and geometry of this system. Although VFD-treated gels have been studied on SANS^15^, this is the first report on real-time SANS measurements. A representation on the setup of the microfluidic platform for the SANS analysis is shown in Fig. 3e, f. Figure 3a–d shows SANS overlays for the real-time VFD data and the post-VFD data of Tween 20 by itself and Tween 20 with fish oil. The differences in the overall scattering intensity for a particular SANS overlay are due to the solvent scattering subtraction step, which is complicated by the experiment setup. Some trends can be observed from the overlays. The Tween 20-only samples all had a weak correlation peak during and after the VFD treatment, with the only noticeable change being after the sample was processed in the VFD at 9000 rpm. The Tween 20 with fish oil samples did not have this correlation peak during the VFD treatment, but after treatment the peak returned; additionally, all fish oil samples exhibited strong low-*q* scattering regardless of the VFD, and the post-7000 rpm sample showed lasting effects.

**Fig. 4:**
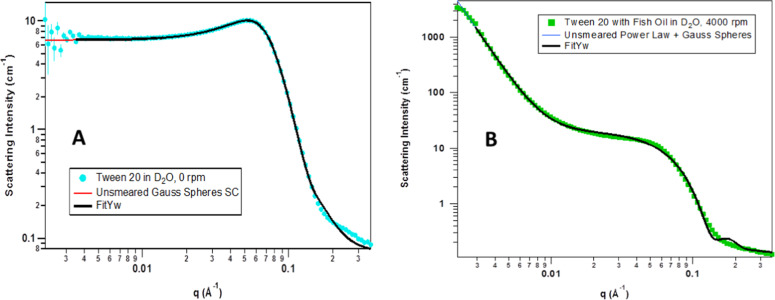
Representative fits to the data from the models described above. **a** Gauss Sphere with screened Coulomb interaction model fitted to the 10 wt% of Tween 20-alone sample at 0 rpm (static), **b** summed Power Law + Gauss Sphere model fitted to the 10 wt% of Tween 20 with 10 wt% of fish oil sample while being sheared at 4000 rpm. The fits to the other data sets shown in the SI are similar in fit quality.

To gain a better understanding of how the size and geometry of the particles changed during and after VFD processing, various models were fitted to the real-time VFD and post-VFD SANS data using non-linear least squares via the NIST macros for Igor Pro (Wavemetrics, Portland, OR, USA)^19^. Different resolution-smeared models were tested on each data set to determine the most appropriate model. Ultimately, a Gauss Sphere with Screened Coulomb interaction model was used for the Tween 20-only samples during and after VFD processing, a summed Power Law + Gauss Sphere with Screened Coulomb interaction model was used for the fish oil samples after VFD, and a summed Power Law + Gauss Sphere model was used for the fish oil samples during VFD treatment. Gauss Sphere reflects the spherical shape and the size distribution of the micelles that can be observed from the epi-fluorescence microscopic image in Fig. 2d. Given that Tween 20 is a non-ionic surfactant, attempts were made to use the hard sphere structure factor with various form factors, such as spheres, oblate and prolate ellipsoids, and core–shell spheres, to model the data; however, none of those were able to fit the correlation peak well. This necessitated the use of the screened Coulomb structure factor, which did fit the correlation peak, thus indicating that the peak was caused by electrostatic repulsions between the micelles. The electrostatic repulsions stem from partial charges on the ether oxygen groups on the ethoxylated polysorbate Tween molecule. Although these partial charges are usually not enough to cause electrostatic interactions, there can be significant electrostatic repulsions if the surfactant concentration and/or the degree of ethoxylation becomes high enough (>20 mM and >20 units, respectively)^20^. In the present systems, the Tween 20 concentration is about 200 mM, making this well above the cutoff for electrostatic interactions to become observable. Example fits to the data can be seen in Fig. 4a, b. The fits to the remaining data sets can be found in the SI.

Table 1 shows the radius and radial polydispersity for the two systems during and after processing at each rotational speed in the VFD. The Tween 20-only samples were not substantially affected by increasing shear in terms of either micelle radius or radial polydispersity, with the micelle radius remaining close to 2.59 nm^21^. Only during the 9000 rpm treatment was there a slight effect on the Tween 20 system, where the micelle radius dropped to 2.57 nm while the micelles became somewhat less polydisperse. Once the shear was removed the micelle sizes remained stable, except the micelles that were sheared at 9000 rpm became slightly smaller and more polydisperse than they were while being sheared. This possibly arises from loss of solvation shell of the micelle under high shear. As the rotational speed increases, the thin film thickness inside the VFD decreases, causing more of the Tween 20 hydrophobic regions to orient toward the oil phase of the emulsions. When fish oil was added, the baseline micelle size at 0 rpm was larger than it was when no fish oil was present, as a result of fish oil being taken up within the micelles. As the systems were sheared at increasing speeds, the micelle sizes grew while the polydispersity dropped, indicating that the system became increasingly homogenized. After they were sheared, the micelle sizes returned to baseline and became similar in polydispersity; however, the system that had been sheared at 7000 rpm ended up having noticeably smaller micelles than baseline (2.29 instead of 2.65 nm) and were more polydisperse, suggesting that the micelles had been pulled apart and then reformed into smaller micelles on average with a greater range in size. This could have caused by strong eddy currents associated with Faraday waves that occur at ca 7000 rpm^9^.

**Table 1:** Sphere radius and polydispersity during and after VFD treatment at each speed for 10 wt% of Tween 20 by itself and 10 wt% of Tween 20 with 10 wt% of fish oil at ratio 1:1.

		Tween 20 alone		Tween 20 with fish oil	
	RPM	Radius (±0.03 Å)	Radial polydispersity (±0.002)	Radius (±0.3 Å)	Radial polydispersity (±0.015)
Real-time VFD	0	25.92	0.27	26.55	0.28
	4000	26.04	0.27	30.31	0.13
	7000	25.93	0.29	30.82	0.12
	9000	25.75	0.22	30.94	0.12
After VFD	0	25.92	0.27	26.55	0.28
	4000	25.86	0.27	26.52	0.27
	7000	25.87	0.26	22.88	0.31
	9000	25.61	0.28	26.60	0.27

**Fig. 5:**
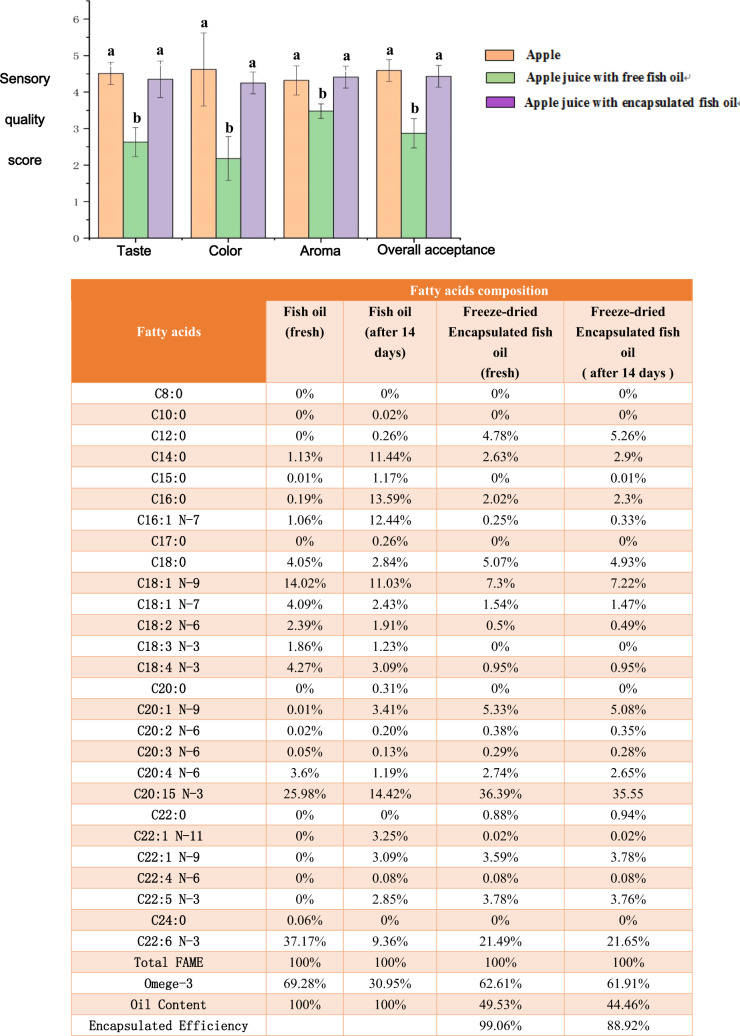
Application of encapsulated fish oil using vortex fluidic device. **a** Sensory evaluation of apple juices with and without fish oil and encapsulated fish oil. **b** Fatty acid profile of fresh fish oil and encapsulated fish oil by VFD processing (speed: 9000 rpm; flow rate: 0.3 mL/min; tilt angle: 45°; temperature: 25 °C) before and after 14 days storage.

Figure 5b shows that the content of omega-3 fatty acids in fresh fish oil purchased from the market is 69.3%. However, after 14 days of storage in an ambient environment, the content of omega-3 fatty acids are reduced to 31.0%, which approximates to a 38% reduction. The results in Fig. 5b show that freeze-dried VFD encapsulated fish oil has little change in the omega-3 fatty acid content after 14 days of storage at ambient conditions, 62.6–61.9%. Relative to as-received fish oil there is only an approximate 7% reduction in omega-3 fatty acid content after VFD processing, with oxidation possibly occurring during the VFD processing associated with the high uptake of gases (oxygen) into the dynamic thin film in the device^8^. Stability of the processed liquid is a prerequisite for the production of encapsulated fish oil powder. Figure 2a, b, e shows the instability of the liquid after homogenization, and therefore, only freeze-drying the liquid after homogenization to form a powder is required. The encapsulation efficiency of the fish oil was also established at 99.1% immediately after VFD processing, and there was only about a 10% drop to 88.9% after 14 days of storage. However, while the encapsulation efficiency dropped approximately 10% after 14 days, the omega-3 fatty acids contents were essentially unchanged (from 62.6 to 61.9%).

An intake of approximately 0.2 g of omega-3 fatty acids per day is recommended^22^. Based on this information, 250 mL of commercial apple juice was enriched with 0.2 g of fish oil and 0.4 g of freeze-dried encapsulated fish oil which contained 0.2 g of fish oil, respectively. This preparation made the concentration of fish oil in enriched apple juice 0.8%(w/v), regardless of VFD-encapsulated fish oil or original fish oil. The quality of the apple juice with different formulations of apple juice with fish oil was evaluated by sensory tests. The average scores with error bars that represent the standard deviation of the sensory evaluations are given in Fig. 5a. There was no significant quality difference between apple juice and apple juice with encapsulated fish oil processed by VFD in terms of taste, color, aroma, and overall acceptance. However, panelists showed a preference for these two samples over the apple juice with free fish oil in every category (Fig. 5a).

**Fig. 6:**
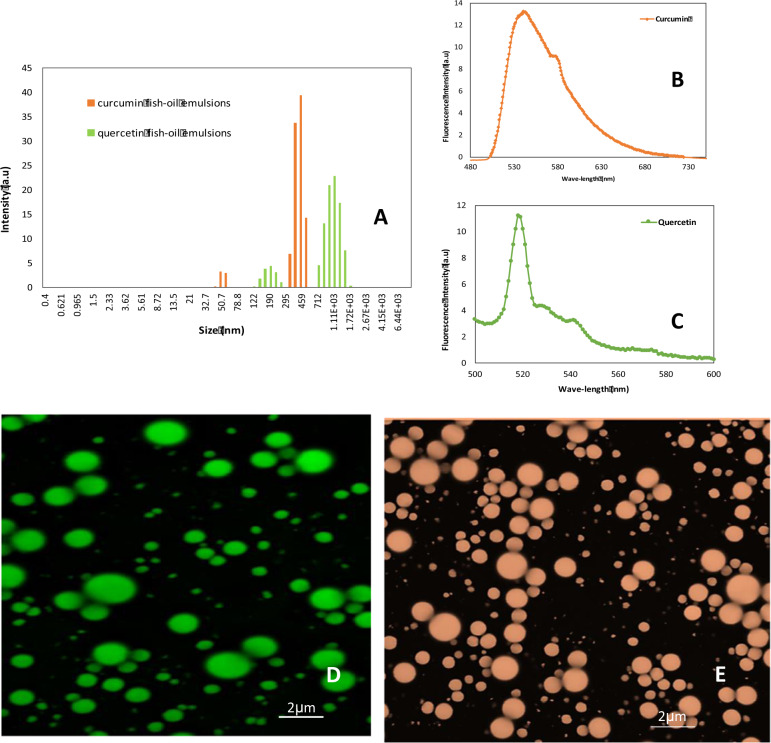
Encapsulation capacity for curcumin and quercetin. **a** Dynamic light scattering (DLS) measuremnets for encapsulated emulsions. **b**, **c** Fluorescence spectra for curcumin and quercetin encapsulated emulsions, respectively. **d**, **e** Confocal micrographic images (wavelength: 420 and 370 nm) of encapsulated fish oil containing curcumin and quecercin, respectively, homogenously suspened in water.

Encapsulations of curcumin and quercetin, as two typical sparingly water and oil-soluble polyphenolic compounds, were formulated using 30 mg of starting raw material, 1 g of fish oil, and 1 g of Tween 20. The conditions of entire encapsulation processing, including VFD operation, were reported in the section of “Encapsulation capacity for curcumin and quercetin” in “Materials and methods” below. The outcome was investigated. The particle size distribution was characterized using DLS. Curcumin-encapsulated emulsions are in a distribution range of 200–500 nm, while the quercetin is indicated at higher distribution range of 700 nm–2 μm (Fig. 6a). Both curcumin and quercetin have characteristic fluorescence signals, Fig. 6b, c, respectively, with fluorescent micrographs observed by confocal microscopy, as shown in Fig. 6d, e, operating at 420 nm for curcumin and 370 for quercetin. Curcumin and quercetin dissolved in the encapsulated fish oil droplets produced green and light orange round droplets homogenously distributed in the water-based system, respectively, with encapsulation capacities as approximately 67.9% and 51.2%, respectively.

## Discussion

The instability of the encapsulated fish oil arises from coagulation of the encapsulated particles^23^. The larger the encapsulated particles, the more they tend to coagulate, and the more unstable they become^23^. Klaypradit and Huang^24^ tested the encapsulation of tuna oil using Tween 80 surfactant and found that, in line with the particle sizes changing from 1.56 to 11.89 μm, the encapsulation stability decreased from 80 to 60%. Previous studies have also reported stable encapsulated fish oil produced by homogenization. However, they either required a multiple-step process, or a high ratio of surfactant to fish oil. Klaypradit and Huang^24^ produced stable emulsions using up to 10:1 of surfactant to fish oil, with the maximum concentration of fish oil at 50 mg/mL. Carla et al.^25^ produced a stable fish oil encapsulation solution with a higher concentration of fish oil at 500 mg/mL, with a lower ratio of surfactant to oil of 2:1. In addition, the process here involved multiple steps, including micelle and buffer preparations prior to encapsulation. Each of these requirements has limitations for industrial applications of encapsulated fish oil, which can be overcome using the VFD. Our initial study^14^ demonstrated that under low concentration in fish oil, VFD processing was able to overcome these two limitations, producing stable encapsulated fish oil in one step, for 20 mg fish oil, 20 mg lipid as surfactant in 10 mL water. The present study further demonstrates the utility of the VFD processing in overcoming the same limitations for producing stable solutions of encapsulated fish oil at much higher concentration (1 g fish oil, 1 g Tween 20 as surfactant in 8 mL water), depending on the choice of surfactant.

SANS measurements of Tween 20 alone and Tween 20 with fish oil were recorded on the Bilby instrument at ANSTO (Lucas Heights, Australia) for increasing speeds (0, 4000, 7000, and 9000 rpm), and measurements were taken again after each speed. The correlation peak indicates the presence of interparticle interactions, which would require a structure factor to be used in the modeling step. The disappearance of the correlation peak in the fish oil samples during VFD treatment is likely to be due to the unincorporated oil interfering with the stabilizing interparticle interactions, which are then able to reform after VFD processing (no shear). The strong low-*q* scattering in the fish oil samples can be attributed to the globules that are large enough to cause cloudiness on the macroscopic scale, which correspond to the approximately 75 nm particles identified using DLS. At higher rotational speed more shear stress (mechano-energy) is applied on the surfactants, lowering the interfacial tension between the oil and water phase, associated with Faraday wave eddies driving interplay of the liquids. (This accounts for enhanced separation of proteins in specific immiscible liquids^17^. As a result, more surfactants adsorb strongly, increasing their emulsifying properties and forming stronger and more compact membranes while under shear. The change in the post-VFD processing at 9000 rpm data curve seen in Fig. 4b likely reflects a slight change in the interparticle interaction. When fish oil is added to the Tween 20 system, the micelle radius increased slightly to 2.65 nm as a result of the fish oil being incorporated into the micelles. While being sheared, the micelles gradually became larger while the radial polydispersity became smaller as the VFD rotational speed is increased, indicating that the VFD homogenized the sample even further and resulted in particles more uniform in size. This effect was, however, not permanent, as seen by the micelle radius and polydispersity returning to baseline in the post-VFD results. After the VFD 7000 rpm treatment, however, the micelle radius dropped to 2.29 nm and the polydispersity increased to 0.31, indicating that the VFD appears to tear the micelles apart to form smaller micelles over a greater size range^26^. This is likely caused by eddy currents that strongly form from Faraday waves at 7000 rpm. It should be noted that the micelle sizes reported by SANS are likely related to the 7–8 nm particles seen by DLS in Fig. 2f, as DLS reports the radius of hydration, which is somewhat larger than the sizes determined by SANS. As the SANS data are on absolute scale, the volume fractions determined by SANS (see SI) also indicate that most of the material in these systems is incorporated in the larger particles identified using DLS, while the small amount of remaining material makes up the smaller micelles identified using SANS. Overall, these results demonstrate that although VFD processing does not generally cause lasting changes to the fully equilibrated system, certain speeds may be effective in causing permanent changes depending on the composition of the system. The mechanoenergy in the VFD is effective in driving systems into nonequilibrium states, as shown in the present system where the nanoparticle-sized fish oil particles are generated as such, and then stabilized by the surfactant.

The oxidation of fish oil was prevented by encapsulation using VFD processing (Fig. 5b). Given the ability to minimize oxidation of the fish oil using VFD processing, there is scope to use the freeze-dried encapsulated fish oil as an ingredient to enrich the content of omega-3 fatty acids in food formulations. In order to demonstrate this, apple juice, a commonly consumed food product, was selected as an example for formulation upgrade by adding it to freeze-dried encapsulated fish oil processed by VFD. The results clearly indicated equal preference of the original apple juice and apple juice with encapsulated fish oil processed by the VFD, over the apple juice with free fish oil. Indeed, a similar study has been reported previously. Tahere et al.^27^ applied encapsulated fish oil into yogurt for the fortification of the omega-3 fatty acids content in yogurt. However, multiple steps were used to form stable fish oil encapsulation, which included preheating, stirring, homogenization, sonication, and storage under nitrogen. In comparison, VFD processing is one step and the thin film microfluidic platform is easy to operate, thereby establishing potential for the fish oil encapsulation in other applications.

Several bioactive compounds are not water-soluble, which restricts their application because they cannot form a homogeneous solution for many water-based food formulations. For example, the antioxidative activities of polyphenolic compounds have been broadly reported. However, many polyphenolic compounds are water-insoluble, although most of the water-insoluble polyphenolic compounds are oil-soluble. Therefore, it was interesting to see if these bioactive compounds could be taken up in fish oil, and encapsulated using a homogeneous single step water-based VFD process. The green (Fig. 6d) and light orange (Fig. 6e) round droplets indicated that the encapsulated fish oil, which included dissolved curcumin (Fig. 6d) and quercetin (Fig. 6e), respectively, were homogenously distributed in the water based systems. The fluorescent micrographs for curcumin and quercetin were taken at each polyphenol’s respective excitation value of 420 and 370 nm. Fluorescence spectrums indicate emission signals for curcumin and quercetin at 540 and 520 nm when excited at their respective excitation values. Emission peaks at 540 and 520 nm for curcumin and quercetin represents lipophilic environments as encapsulated particles in the fish oil emulsions. The particle size for curcumin and quercetin are in the range 200–500 nm and 700 to 2 μm which is evident from confocal micrographs; the smaller particles at 50 and 100 nm presumably aggregated curcumin and quercetin particles not encapsulated in the emulsions.

The encapsulation of water-insoluble polyphenols has previously been reported, but it either required multiple wall-materials (surfactants), such as chitosan and tripolyphosphates, for encapsulation involving multiple step processing^28^ or the use of expensive equipment, such as a electrohydrodynamic atomizer^29^. VFD processing is effective and the device is relatively inexpensive.

In summary, a facile process, operating under continuous flow has been developed using a VFD, for encapsulated particles, 100–200 nm in diameter. This VFD simplifies the processing procedure of encapsulation into a time- and cost-saving one-step process devoid of any organic solvents, which is in contrast to the conventional homogenization process, which is inherently complex and involves multiple steps and the use of organic solvents. VFD encapsulation minimized the oxidation of the encapsulated fish oil, from 38.3% (approximate 69.3–31.0%) to 7.3% (approximate 69.3–62.0%). The encapsulated particles are a storage compartment for omega-3 fatty acid in water-based food products, demonstrated for apple juice, without changing their sensory values. The encapsulation capacity of curcumin and quercetin was 67.9% and 51.2%, respectively, and the findings provide a model for producing homogenous suspensions of water-insoluble bioactive compounds in general. Both VFD processes of encapsulated fish oil and simultaneously encapsulating water-insoluble bioactive polyphenols was scaled-up from 10 to 200 mL, with the solutions found to have the same overall characteristics. This study not only presents a new alternative approach as an affordable one process but also for directly scaling up any fish oil process, for a variety of applications in food processing.

## Methods

### Materials

Fish oil enriched with omega-3 fatty acids was provided by Chemist Warehouse (Australia). Tween 20 was purchased from Sigma-Aldrich (NSW). Milli-Q water was used throughout the preparations of the encapsulated fish oil.

### Fish oil encapsulated with Tween 20

The encapsulation process using VFD were operated according to He et al.^30^ with modifications. Encapsulation of fish oil, the bioactive ingredient, used a mixture of Tween 20, as a surfactant, and water. Oil-in-water encapsulations had a surfactant-to-oil ratio of 1:1 (w:w) with a total concentration of 0.2 g/mL. Briefly, 10 mL of the Tween 20 oil suspension in water was premixed as an emulsion (1 g of oil, 1 g of Tween 20, and 8 mL of water) and then added to a borosilicate glass tube (20 mm OD, 17 mm ID) in the VFD through jet feeds with the tube rotating at 9000 rpm, at a flow rate of 0.3 mL/min, with the tilt angle of the tube at 45°, and the device operating at room temperature. This condition was deemed as the optimal condition for encapsulation of fish oil after systematically varying the rotational speed (4000–9000 rpm), flow rate (0.3–0.5 mL/min), and tilt angle (30°–60°). The traditional homogenization method was also applied for comparison with VFD processing. Indeed, the oil to surfactant ratio of 1:2 has a slightly better effect, but the implication of this ratio would cause more surfactant to be used in production. This would increase the production cost. Therefore, the conditional optimization was chosen to be 1:1. Homogenization, the conventional method for encapsulation, served as a control for the present study, using the same concentrations that were used for VFD processing with the homogenizer (T25 digital ULTRA-TURRAX) operating at 13,500 rpm for 10 min, at 25 °C. The VFD-mediated encapsulated fish oil solution was freeze-dried for further use.

### DLS technique

The particle-size distribution of VFD-encapsulated fish oil and the polydispersity index were determined at 25 °C using DLS (Nano ZS90, Malvern instruments, Worcester, UK) operating with a He–Ne 633 nm wavelength laser and a detector angle of 173°. The Malvern zeta sizer instrument measured the time-dependent fluctuations of light scattered based on the particle sizes.

### Epi-fluorescence microscopy

Images of encapsulated particles were recorded using an epi-fluorescent microscope with a monochrome camera (NIKON DS-Qi1Mc), a Cool LED pE300, and appropriate filter cubes. A 20× long-working-distance (20× amplification) objective with an exposure time of 100 ms and a frame rate of approximately 10 fps was applied. Images taken by the microscope were analyzed using the program ImageJ.

### Fatty acid profile of fresh fish oil and VFD-encapsulated fish oil before and after 14 days

Fresh fish oil and freeze-fried VFD-encapsulated fish oil before and after 14 days of storage were subjected to fatty acid profile measurements following the method described by He et al.^31^, with slight modification. Extracted fats were converted to fatty acid methyl esters (FAME), separated, and then measured on a Hewlett-Packard 6890 gas chromatograph equipped with a 50-cm capillary column (0.32 mm internal diameter SGE; Victoria, Australia) coated with 70% cyanopropyl polysilphenylene-siloxane (BPX-70; 0.25-lm film thickness), which was fitted with a flame ionization detector. Helium was the carrier gas (flow rate 60 mL min^−1^), and the split ratio was 20:1. The injector temperature was set at 250 °C and the detector temperature at 300 °C. The initial oven temperature was 140 °C and was programmed to rise to 220 °C at 5 °C/min and held for up to 3 min. FAMEs were identified based on the retention time of standards obtained from Nucheck Prep Inc. (Elysian, MN, USA) using the Chemstation software. An external standard was analyzed and used for calibration.

### Encapsulation efficiency

The VFD-mediated encapsulated fish oil was freeze-dried, and the oil content of the fish oil particles before and after 14 days of storage was measured according to He et al.^31^. Encapsulation efficiency was calculated as the percentage of the oil content of the freeze-dried VFD-mediated encapsulated fish oil in the fish oil starting material (1 g).

### Enrichment of apple juice with nano-encapsulated fish oil

An intake of approximately 0.2 g of omega-3 fatty acids per day is recommended^22^. Based on this information, 250 mL of commercial apple juice was enriched with 0.2 g of fish oil and 0.4 g of freeze-dried encapsulated fish oil which contained 0.2 g of fish oil, respectively. This preparation made the concentration of fish oil in enriched apple juice 0.8% (w/v), regardless of VFD-encapsulated fish oil or original fish oil. Samples were stirred with a magnetic stirrer for 1 h.

### Sensory tests

Sensory tests were conducted according to Zeng et al.^32^ with slight modifications. Forty volunteers were recruited according to standard procedures. The sensory evaluation was performed in private booths equipped with Sensory Management System hardware (2006) and computerized sensory software (Sensory Integrated Management System, Morristown, NJ, USA). The volunteers received 1 h of training in discrimination testing, followed by five rounds of practice triangle testing. Volunteers who scored poorly more than twice during these five rounds of practice tests were eliminated from the panelist pool. The number of panelists who passed the practice triangle test was 20. Sensory evaluation consisting of color, taste, aroma, and overall acceptance were based on hedonic scales (1: dislike extremely; 5: like extremely). Each sample was scored individually, and the samples were presented to the panelists in individual plastic containers. The samples were coded and randomly presented to the panel group at each session. Water was presented to rinse their palate between samples^33^.

### Encapsulation capacity for curcumin and quercetin

Encapsulations were formulated using 30 mg of starting raw material (curcumin or quercetin), 1 g of fish oil, and 1 g of Tween 20. Briefly, 10 mL of a 30 mg starting raw material, 1 g of fish oil, and 1 g of Tween 20 suspension in water was premixed as an emulsion, then introduced into a borosilicate glass tube (20 mm OD, 17 mm ID) in the VFD through jet feeds with the tube rotating at 9000 rpm, at a flow rate of 0.3 mL/min, with the tilt angle of the tube at 45°, and the device operating at room temperature. The product was collected, then centrifuged at 3000 rpm for 20 min. The precipitate after centrifugation, which was non-encapsulated starting raw material, was collected and dissolved in 200 mL of 80% (v/v) ethanol for the measurement of absorbance at a wavelength of 420 nm. The standard curve with the *x*-axis of the weight of dissolved starting raw material in 200 mL of 80% (v/v) ethanol and *y*-axis of absorbance was generated. The weight of precipitated starting raw material was determined according to this standard curve. The encapsulation efficiency of curcumin and quercetin was calculated according to the following equation:

$${\mathrm{Encapsulation}}\,{\mathrm{capacity}}\,\left( \% \right) = \frac{{30\,{\mathrm{mg}}\,{\mathrm{of}}\,{\mathrm{original}}\,{\mathrm{material}} - {\mathrm{the}}\,{\mathrm{weight}}\,{\mathrm{of}}\,{\mathrm{precipitated}}\,{\mathrm{material}}}}{{30\,{\mathrm{mg}}\,{\mathrm{of}}\,{\mathrm{original}}\,{\mathrm{material}}}} \times 100.$$

### Confocal microscopy technique

Encapsulations were formulated using 30 mg of curcumin or quercetin, 1 g of fish oil, and 1 g Tween 20. Briefly, 10 mL of 30 mg curcumin or quercetin, 1 g of fish oil, and 1 g of Tween 20 suspension in water was premixed as an emulsion, then introduced into a borosilicate glass tube (20 mm OD, 17 mm ID) in the VFD through jet feeds with the tube rotating at 9000 rpm, at a flow rate of 0.3 mL/min, with the tilt angle of the tube at 45°, and the device operating at room temperature. The product was collected, then centrifuged at 3000 rpm for 20 min. The supernatant was collected for confocal microscopy according to the method described by Coklin et al.^34^ with slight modifications. Briefly, 25 μL of the supernatant was dripped onto the slide and covered with a glass slide, then dried overnight. Nail varnish was applied to seal the ends of the glass slide after drying overnight. A confocal laser scanning microscope was then applied to observe the microstructure of the encapsulated particles, with the wavelength at 420 nm for curcumin and 370 nm for quercetin, which are the excitation wavelengths of curcumin and quercetin, respectively.

### Fluorescence spectroscopy measurements

All the fluorescence measurements were carried out with Cary Eclipse Fluorescent Spectrophotometer, Agilent technologies using quartz cells of path-length 10 mm. Both excitation and emission band slits were fixed at 10 nm and the scan rate was selected at 1800 nm/min. The excitation wavelength was selected at 425 nm, while emission spectra were collected in the range of 450–600 nm for curcumin and the excitation wavelength for quercetin was 440 nm while the emission was collected in the range 500–600 nm.

### Statistical analysis

The measurements of emulsifying stability and encapsulation capacity for curcumin and quercetin were carried out in triplicate. The measurements of sensory tests were carried out for 20 replicates (20 qualified panelists). Data were presented as the mean with standard deviation. One-way analysis of variance (ANOVA) and least significant difference (LSD) using MINITAB Statistical Software v15 were applied in the statistical analysis. The significance was judged statistically by the *F* value at a probability (*p*) below 0.05.

### Small-angle neutron scattering

Solutions of 10 wt% Tween 20 were prepared in D_2_O at room temperature and allowed to equilibrate for 24 h, while 10 wt% Tween 20 with 10 wt% fish oil were mixed together in D_2_O using a magnetic stirrer. D_2_O was used as the solvent to improve the contrast between the continuous and dispersed phases. SANS scans were taken at 0 rpm (static) to serve as a baseline of comparison for the higher speeds. Depending on the VFD speed, different volumes of sample were added to a quartz VFD tube to ensure that the thin film would not go too high up the side of the tube. For the real-time 4000 rpm scans, 2 mL of sample was added to the sample tube, while 1 mL was used for the 7000 and 9000 rpm runs.

SANS experiments were carried out at Bilby (small-angle neutron scattering time of flight) (ANSTO)^35^. The VFD microfluidic device was mounted on an *xyzθ* goniometer stage. The stage was adjusted with respect to the neutron beam such that the beam-center was placed 3 cm from above the base of the tube, schematically shown in Fig. 3. For all experiments, a quartz tube is used instead of normal borosilicate glass tube to reduce background for SANS. All the quartz tubes possess 20 mm OD. An aluminum shield was put on the VFD to protect the tube from any outside impacts. Slits were cut from the sides of the shield and covered with aluminum foil in order to reduce the loss of beam intensity due to scattering off of or absorption into the aluminum. Sample detector distances of 1.3, 12.0, and 20.0 m were used to achieve a *q*-range of 0.00216 < *q* < 0.38125 Å^−1^, where *q* = (4*π*/*λ*)sin*θ*, 2*θ* is the scattering angle, and λ is the wavelength. The acquisition times for all experiments were 60 min. each. Mantid software^36^ was employed for all data reduction. The 2D data were corrected for background scattering, empty tube scattering, solvent scattering using D2O blanks processed at each VFD speed (0, 4000, 7000, and 9000 rpm), and detector sensitivity, and then set to absolute scale based on the beam transmission. The reduced 2D data were then radially averaged to obtain the 1D data. The 1D data was analyzed in Igor Pro (WaveMetrics, Portland, OR, USA) using the analysis macros developed at NIST^19^.

The general equation describing the scattering for particulate systems is $I\left( q \right) = \frac{N}{V}\left( {\rho _1 - \rho _2} \right)^2P\left( q \right)S\left( q \right)$, where *N* is the number of scatterers, *V* is the total sample volume, *ρ*_*n*_ is the scattering length density (SLD) for the solvent phase or particle phase and (*ρ*_1_ − *ρ*_2_)^2^ is the contrast factor, *P*(*q*) is the form factor that describes the particle geometry, and *S*(*q*) is the structure factor describing the inter-particle interactions. Here, the form factor used was spheres with a Gaussian polydispersity term for the radius. This form factor is well-known and can be found in the SANS Toolbox^37^ for example, which also provides a great deal of information on the SANS technique and other models in general. The presence of correlation peaks in the scattering data indicated that there were interparticle interactions, necessitating the use of a structure factor. The strong correlation peak could not be modeled well by a hard-sphere interaction, but the screened Coulomb interaction was able to model the peak suggesting that the scattering particles were charged and repelling each other electrostatically. The repulsive potential, *U*(*r*), for the screened Coulomb interaction is

$$U\left( r \right) = \left\{ {\begin{array}{*{20}{l}} {\infty ,} & {r \,\le \,\sigma } \\ {\left( {z^2/4\pi \varepsilon \varepsilon _0\left( {1 + \frac{{\kappa \sigma }}{2}} \right)^2} \right)\frac{{e^{ - \kappa \left( {r - \sigma } \right)}}}{r},} & {r \,>\, \sigma } \end{array}} \right.,$$

where *r* is the center-to-center distance between the ionic particles, *σ* is the diameter of the particle, *z* is the charge of the particle, *ε* is the dielectric constant of the solvent, *ε*_*o*_ is the permittivity of free space, and *κ* is the Debye–Hückel inverse screening length. By using the mean spherical approximation to solve the Ornstein–Zernike equation for particulate systems, the direct correlation function *c*(*r*) becomes *c*(*r*) = −*βU*(*r*), where *β* = 1/(*k*_*B*_*T*) is the inverse temperature in energy units. This analytical solution was developed by Hayter and Penfold^38^ and further expanded by Hansen and Hayter^39^ for instances where *S*(*q*) is needed to analyze experimental data. Further discussion of this structure factor can be found in those references.

## Data Availability

The data that support the findings of this study are available from the corresponding author upon reasonable request.
